# Research on trajectory control technology for L-shaped horizontal exploration wells in coalbed methane

**DOI:** 10.1038/s41598-024-60550-4

**Published:** 2024-05-18

**Authors:** Xiugang Liu, Zaibing Jiang, Yi Wang, Haitao Mo, Haozhe Li, Jianlei Guo

**Affiliations:** 1 China University of Mining and Technology, Beijing, 100083 China; 2 China Coal Research Institute, Beijing, 100013 China; 3 Xi’an Research Institute Co. Ltd., China Coal Technology and Engineering Group Corp., Xi’an, 710077 China

**Keywords:** Coalbed methane (CBM), Short-radius wells, Trajectory control, Pilot hole, Two-dimensional resonance exploration, Azimuth gamma logging while drilling (LWD), Energy science and technology, Engineering

## Abstract

Horizontal wells have significant advantages in coal bed methane exploration and development blocks. However, its application in new exploration and development blocks could be challenging. Limited geological data, uncertain geological conditions, and the emergence of micro-faults in pre-drilled target coal seams make it hard to accurately control the well trajectory. The well trajectory prior to drilling needs to be optimized to ensure that the drilling trajectory is within the target coal seam and to prevent any reduction in drilling ratio (defined here as the percentage of the drilling trajectory in the entire horizontal section of the well located in the target coal seam) caused by faults. In this study, the well trajectory optimization is achieved by implementing the following process to drill pilot hole, acquire 2D resonance, and azimuthal gamma logging while drilling. The pilot hole drilling can obtain the characteristic parameters of the target coal seam and the top and bottom rock layers in advance, which can provide judgment values for the landing site design and real-time monitoring of whether the wellbore trajectory extends along the target coal seam; 2D resonance exploration can obtain the construction of set orientation before drilling and the development of small faults and formation fluctuations in the horizontal section, which can optimize the well trajectory in advance; the azimuth gamma logging while drilling technology can monitor the layers drilled by the current drill bit in real time, and can provide timely and accurate well trajectory adjustment methods.The horizontal well-Q in the Block-W of the Qinshui Basin was taken as a case study and underwent technical mechanism research and applicability analysis. The implementation of this new innovative process resulted in a successful drilling of a 711 m horizontal section, with a target coal seam drilling rate of 80%. Compared to previous L-type wells, the drilling rate increased by about 20%, and the drilling cycle shortened by 25%. The technical experience gained from this successful case provides valuable insight for low-cost exploration and development of new coalbed methane blocks.

## Introduction

**Figure 1:**
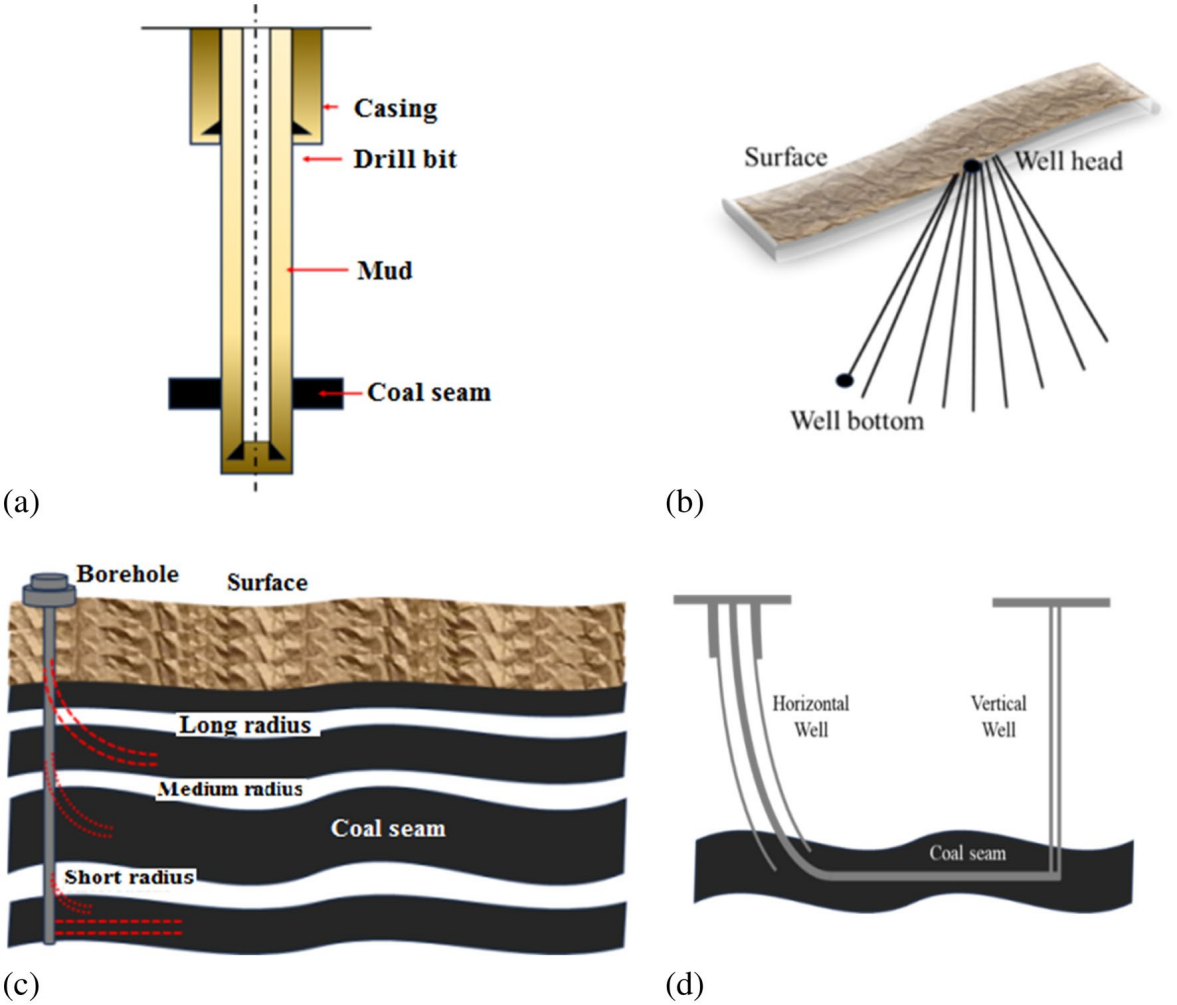
Illustration of well types; (**a**) Vertical well, (**b**) Cluster well, (**c**) Horizontal Well, and (**d**) Horizontal L-Shaped well with a vertical well forming a U-Shaped well.

Coal Bed Methane (CBM) is found in many parts of the world, and is considered as a clean and abundant source of energy^1–3^. In general, CBM wells mainly include three types; vertical, cluster and horizontal wells. The cluster and horizontal wells belong to directional wells. Moreover, horizontal wells could be further classified into; V-, U- and L-shaped wells. Which in turn could also be divided according to their radius, and branches. Figure 1 below provide an illustration for some of these wells.

In the development of CBM wells, L-shaped, U-shaped and multi-branch horizontal wells are usually used for new exploration and development blocks (defined here as new fields or area blocks in the oil and gas industry)^4–6^. However, complex formation structure, and small faults development have made it an extremely challenging task to achieve high output from newly developed CBM wells^7^. For instance, U-shaped wells (a well type in which a vertical well and a horizontal well are connected in the same target layer) face huge difficulties in accurate docking along the coal seam and have limited benefits in the presence of multiple faults in the horizontal Section ^8^. Similarly, the applicability of multi-branch horizontal wells is poor, especially in complex stratigraphic structures and fault development of the block^9^.

On the other hand, L‑shaped horizontal wells are often adopted as the main type of wells for exploring and developing CBM in new blocks. The L-shaped horizontal wells exhibit uncomplicated drilling prerequisites, demonstrate a low probability of wellbore collapse or obstruction, and facilitate subsequent access for maintenance of the initial wellbore^10^. However, the drilling process of these wells are not free of challenges. L-shaped wells have a high requirement for wellbore trajectory control, and they are usually difficult to achieve one-time “soft landing” and ultra-long horizontal segment footage^10^. In addition, drainage equipment and method are another key restriction for the promotion and application of this type of well^11^. For example, reported completion data from several exploration wells indicated that the drilling ratio along the coal seam of the actual trajectory is less than 60%. The drilling cycle is nearly two months, and gas production is low^11^. Table 1 illustrates a tabulated analysis of the applicability and challenges associated with different well types in exploration blocks characterized by complex geological formations and the presence of micro-faults.

**Table 1:** Applicability and challenges of different well types.

Well type	Applicability and challenges
Vertical well	Applicable for later-stage low-yield coalbed development
U-shaped well	Challenging precision docking with coal seams; lower coal seam drilling encounter rate in cases of multiple fault development within the horizontal well section
Multi-branch horizontal well	Suitable for well-structured geological conditions; challenging to ensure that each branch hole penetrates the target coal seam, leading to uncertainty in drilling encounter rates
L-shaped horizontal well	Simple drilling requirements; minimal risk of borehole collapse and blockage post-production; enables well-cleaning and original wellbore re-entry for maintenance

Various methods have been used to improve the drilling ratio, by improving the trajectory control. These methods, shown in Table 2, include: geological guidance technology of adjacent well data, electromagnetic waves, natural gamma measurement, and three-dimensional seismic exploration technology. However, each method has its own limitations, such as high costs, difficulty in obtaining gamma values in specific directions, and signal loss when applied to drilling in complex formations^12,13^.

**Table 2:** Trajectory Control Methods: Advantages and Disadvantages.

Method	Advantages	Disadvantages
Geological guidance with adjacent well data	Utilizes existing well data for guidance	Limited by data availability and quality
Electromagnetic wave guidance	Offers real-time guidance	Affected by subsurface conditions
Natural gamma measurement	Provides valuable subsurface data	May not offer a comprehensive picture
Three-dimensional seismic exploration	Offers high-resolution imaging	Expensive and requires specialized equipment

This study delves into trajectory control methods for Horizontal wells within Coalbed Methane (CBM) exploration and development blocks. The approach involves the utilization of pilot holes to determine the characteristics of the target coal seam and the surrounding upper and lower rock layers based on the magnitude of gamma values. This information serves as a predictive identification of marker layers, allowing real-time control and adjustment of the drilling trajectory within the target coal seam. This methodology enables the identification of whether the drilling trajectory is presently positioned within the target coal seam, the roof rock layer, or the floor rock layer. Additionally, a two-dimensional resonance exploration technology is employed for geological structure and fault detection prior to drilling, enabling pre-drilling trajectory optimization. Furthermore, azimuth gamma logging technology is utilized for real-time monitoring and correction of the drilling trajectory's horizontal positioning during the drilling process. Using L-shaped Short-Radius Well-Q in Block-W of the Qinshui Basin as a case study, a comprehensive assessment of the combined effectiveness of these three methods is conducted. Simultaneously, the research delves into the technical mechanisms and applicability analysis. This exploration of the technical mechanisms aims to enhance the understanding of the functions of these methods, their application conditions, and the analysis and utilization of their technical effects.

## Trajectory control methodology

### Pilot hole drilling

#### Construction background and reasons

The area formation structure and faults nature could be obtained by two-dimensional seismic data. Seismic surveys and exploratory drilling in the area could provide a good indication on the coal seam actual depth, coal seam distribution, layers, belts and interbeds. For the geological conditions of developing new blocks, such as less drilling data, less seismic exploration data, complex formation structure and micro-fault development, etc., before drilling, it is imperative to obtain the key parameters of the target coal seam, including its lithology, gas-bearing capacity, gamma value, etc., along with those of the rock layers above and below it. This will allow for the determination of the precise horizon of the coal seam and provide technical support for real-time monitoring and well trajectory control along the target coal seam. To achieve this, it is necessary to design and implement a pilot hole drilling program to obtain the characteristic parameters of the target coal seam and the surrounding strata^14,15^.

#### Pilot hole construction design

**Figure 2:**
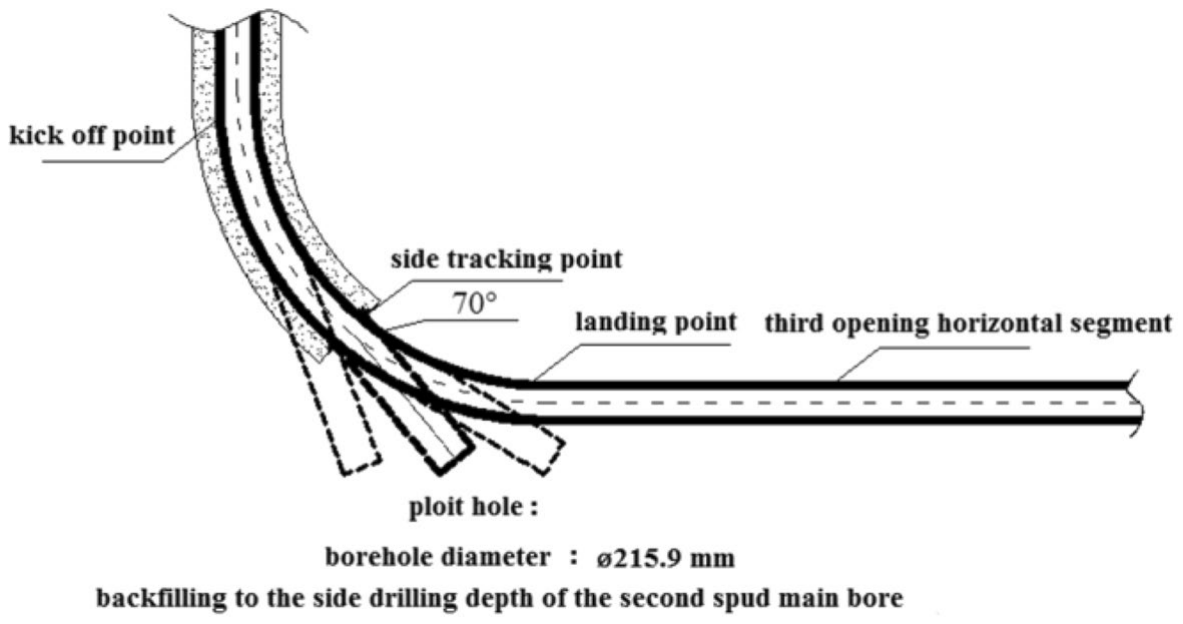
Optimization of pilot hole scheme.

Once the goal of layer identification is achieved, the next step is to backfill and sidetrack the pilot hole to open branches and land according to the actual occurrence of the coal seam. To ensure the effectiveness of the pilot hole guidance in subsequent construction, it is advisable to minimize the distance between the coal-seem top point (the point where the drilling trajectory first drills into the target coal seam) and the landing point by increasing the well angle of inclination. Conversely, in order to enhance the construction efficiency of the pilot hole, it is preferable to keep the depth of the pilot hole to a minimum, which is indicated by a small well angle of inclination (70 degrees). Figure 2 illustrates this concept.

Taking into account the underlying reasons and background for constructing a pilot hole, as well as the difficulty of side-tracking and the efficiency of construction, a comprehensive plan has been developed. The plan involves drilling the pilot hole at a steady angle of approximately 70° until the bottom of the target coal seam is reached.

### Two-dimensional resonance exploration

#### Resonance exploration mechanism

**Figure 3:**
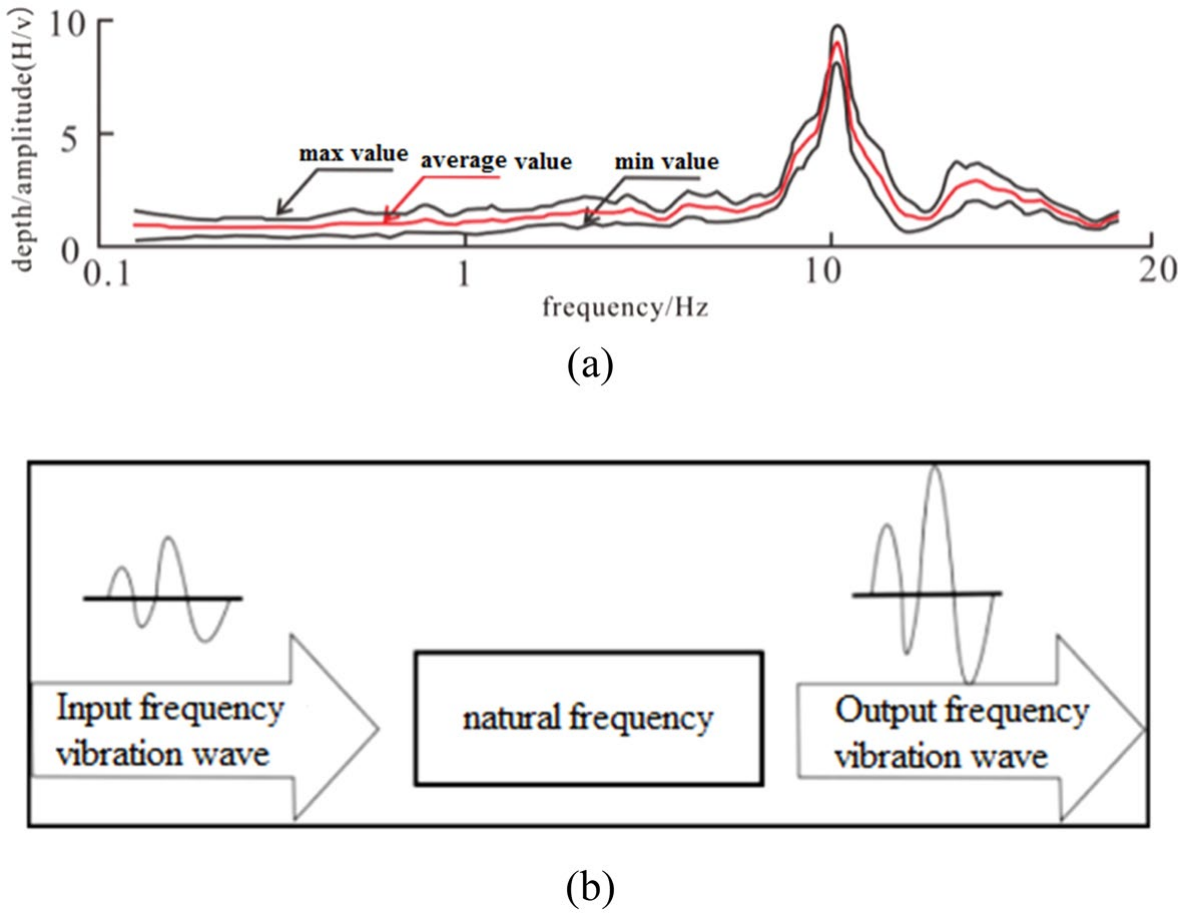
(**a**) Typical resonance curve of seismic wave (**b**) self-excite resonance to vibration.

The seismic wave frequency resonance exploration technology is a novel geophysical exploration method that utilizes the frequency resonance principle prevalent in nature to investigate underground geological formations^16–19^. This technique enables the acquisition of geometric attributes of subsurface structures, such as fractures and faults. Figure 3 illustrates a typical resonance diagram of a seismic wave.

Resonance exploration technology boasts numerous advantages, including high sensitivity to density changes, exceptional vertical and horizontal resolution, and an exploration depth of up to 5000 m. Additionally, this technology can be acquired and processed passively, making it an economical and straightforward exploration method^20^.

#### Analysis of technical applicability

At this stage, the analysis of the existing two-dimensional seismic data in the exploration block would indicate the geological structure of the target coal seam in the block. In addition, it will reveal fault’s locations beside faults development status. The pilot hole drilling can accurately obtain the actual depth of the target coal seam and the characteristic parameter values of the target layer, as well as the roof and floor, but conventional means cannot predict structural conditions such as the development of micro faults in the horizontal section of the drilling along the designated direction. This increases the difficulty of well trajectory control and makes it challenging to ensure the coal seam drilling ratio. However, the two-dimensional resonance exploration technology can be used to infer the development of small faults in the horizontal section drilled along the specified direction by interpreting the resonance image. This enables the optimization of the well trajectory in advance to control the actual drilling trajectory and improve the drilling rate of the target coal seam.

### Azimuth gamma control technology

#### Working principle of azimuth gamma

The azimuth gamma logging tool is utilized to measure the width of gamma ray energy level^21–23^. The scintillation counter captures gamma rays from the stratum, and azimuth gamma logging while drilling offers unique advantages^24,25^. Firstly, it enables real-time calculation of the strata's apparent dip angle. It is convenient to calculate the apparent dip angle of the strata by utilizing the azimuth gamma data. The apparent dip angle at the current position can be obtained as long as it is required to cross an interface. The formula for calculating the apparent dip angle using the azimuth gamma^26^ is as follows:

1 $$\mathrm{\alpha }\approx {\text{arctan}}\left({\text{D}}/\Delta {\text{d}}\right)+\upbeta -90^\circ $$

where α is the apparent strata dip; *D* is the well diameter; *Δd* is the distance between the upper and lower gamma value change points; β is the well deviation angle.

**Figure 4:**
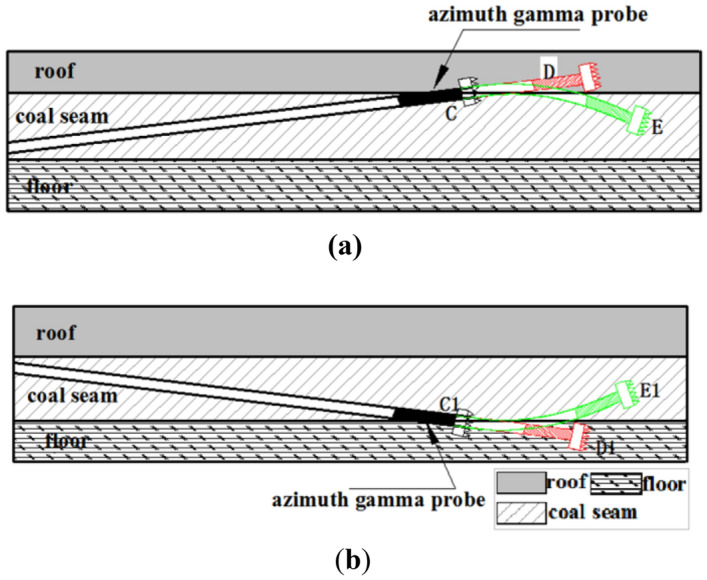
Trajectory control based on azimuth-while-drilling gamma logging. (**a**) Coal seam drilled out from the roof. (**b**) Coal seam drilled out from the floor.

Second, measuring the natural gamma value in a specific direction. By transmitting up and down gamma data in real-time, it becomes possible to accurately determine the positions of different formation interfaces^27,28^. This information can then be used to ensure that the trajectory of the control well is precisely aligned with the target coal seam after drilling is complete. The specific process involved is illustrated in Fig. 4.

The drilling process in the horizontal section along the coal seam is susceptible to deviate from the target due to increased drilling pressure or the impact of the formation structure. The strata above and below the coal seam are usually mudstone or carbonaceous mudstone. When using azimuth gamma logging during drilling, the upper gamma value first increases, followed by the lower gamma value, indicating that the drilling has exited the coal seam roof at point C in Fig. 4a. When the upper and lower gamma values become similar, it suggests that the drilling has left the layer, as shown at point D in Fig. 4a. To correct the inclined drilling control track deviation, the trajectory correction process is initiated when drilling to point C using azimuth gamma measurement, as demonstrated at point E in Fig. 4a. Similarly, when the lower gamma value increases first and the upper gamma value increases later, it indicates that the drilling trajectory is exiting the coal seam floor at point C1 in Fig. 4b. When the upper and lower gamma values become similar, the drilling has left the layer, as shown at point D1 in Fig. 4b. To correct the incremental drilling control track deviation, the trajectory correction process is initiated when drilling to point C1, as illustrated at point E1 in Fig. 4b.

In terms of technical applicability, conventional natural single gamma logging technology cannot accurately determine the bit's position once it leaves the coal seam, making it challenging to provide precise corrective measures. This issue is particularly problematic wherever the geological structure of the target coal seam is complex, micro faults are developed, and the coal seam is thin. To ensure the penetration ratio of the target coal seam and ensure the safety of underground construction, azimuth gamma logging while drilling technology can be utilized. This technology allows for the real-time monitoring of the current drilling horizon and provides effective guidance during construction. As a result, the drill bit can efficiently drill into the coal seam, maximizing the penetration ratio of the target coal seam.

#### Technical applicability analysis

In the second drilling operation, if the targeted coal seam is complex due to its thinness or the presence of micro-faults, it will be very challenging to accurately determine the position of the drilling bit after it exits the coal seam. Therefore, it will be necessary to use azimuth gamma logging while drilling. This technology enables the real-time monitoring of the drilling bit's current horizon, guiding the construction process and ensuring that the bit drills to the maximum extent possible within the coal seam.

## Trajectory control technology and case study

### Geological setting

In this study, the short radius, well-Q in Block-W of the Qinshui Basin is taken as an example. Based on the most recent exploration wells drilled in Block-W of Qinshui Basin, the geological horizons have been revealed. The strata in the block, from bottom to top, consist of Paleozoic Ordovician, Carboniferous, Permian, Mesozoic Triassic, Jurassic, and Cenozoic Quaternary. The stratum near Well-Q has a general inclination from northeast to northwest, and Coal Seam no.15 is the development target stratum. The coal seam is located in the lower part of the Taiyuan Formation and has a simple structure. It is a thick coal seam that is stable and easy to drill throughout the area and generally contains 0–2 layers of dirt shale. The effective thickness of the coal seam ranges from 0 to 5.30 m, with an average of 3.39 m. It is thicker in the east and thinner in the west. However, there is one exploration well in the block that did not drill into Coal Seam no.15, possibly due to fault interference resulting in the loss of the coal seam. The coal seam deposit depth ranges from 728 to 2002 m, with an average of 1479 m. The depth is shallow in the southeast of the block and gradually deepens towards the northwest. Due to the influence of the stratum tendency (Stratum dip), the depth of the coal seam reaches over 1500 m in the west^14^. The roof lithology of the coal seam mostly consists of sandy mudstone, mudstone, siltstone, and fine sandstone, while the floor is mostly sandy mudstone, mudstone, and siltstone.

### Wellbore structure

Designing an optimized wellbore structure can greatly improve drilling efficiency and safety by reducing annular pressure loss and back pressure (the drilling tool back pressure phenomenon), especially for long well sections. In the case of Well-Q, the wellbore structure was designed with a three-opening sections to ensure gas production of the coal seam during subsequent fracturing development. The first section seals the formation prone to collapse and leakage in the upper part of the primary casing, creating a safe drilling environment for the second well section. The second section seals sandstone, mudstone, and sandy mudstone intervals at the upper part of the coal seam, with the second well section casing obliquely drilled to a depth of no less than 3 m from the target coal seam no.15.

**Figure 5:**
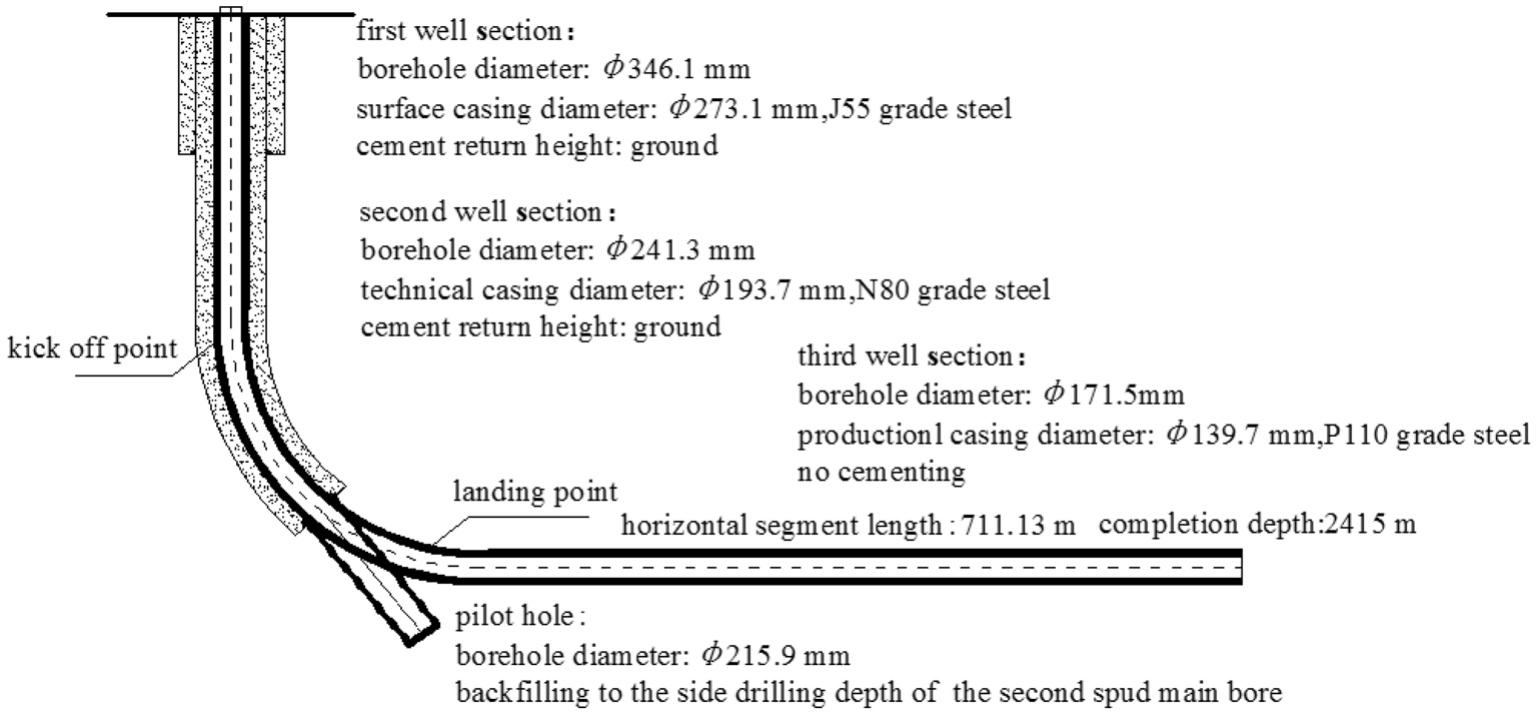
Well structure.

The third section extends along coal seam no.15 and runs casing to form a stable gas production channel to prevent coal seam collapse in the horizontal section due to the influence of multiple factors such as fracturing in the later stage. Prior to drilling the second well section of the main borehole, pilot hole drilling was carried out to obtain relevant geological parameter information of the target coal seam and the adjacent marker bed. Specific design parameters and requirements are as follows:

1. In the first well section, a ø 346.1 mm drill bit was used to drill into the stable bedrock for 30 m. J55 grade steel ø 273.1 mm surface casing was then lowered and cementing cement slurry returned to the surface.
2. In the second well section, a ø 241.3 mm drill bit was used to drill to the roof of the target no.15 coal seam and then the drilling was stopped. The landing point was determined based on the lithology of the roof of the coal seam and the actual drilling process. N80 grade steel ø 193.7 mm technical casing was run to 3–5 m above the roof of the coal seam. Through variable density cementing process, high-density cement slurry was used to return to 300 m above the roof of Coal Seam no.15, while low-density cement slurry returned to the surface.
3. The third well section was drilled with a ø 171.5 mm drill bit. After entering the target coal seam no.15, the drilling followed the coal seam. Upon reaching the designed well depth, P110 grade steel ø 139.7 mm production casing was run, and the well was completed without cementing.
4. The pilot hole was drilled with a ø215.9 mm bit, and the inclination angle stabilizing drilling crossed the floor of the target coal seam for tens of meters. Subsequently, the bit was backfilled with pure cement slurry to the side drilling depth of the second well section. The specific wellbore structure is shown in Fig. 5.

### Case study: well-Q design optimization

**Figure 6:**
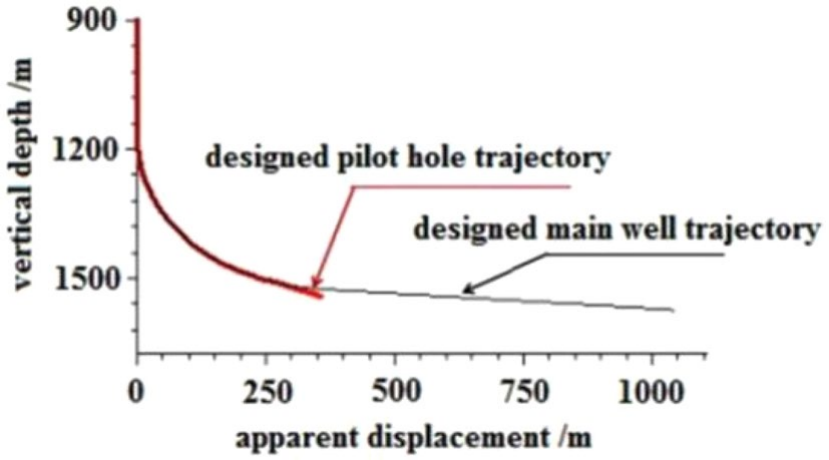
Design trajectory of pilot hole and main hole.

Using Well-Q as a case study, the pilot hole trajectory design included the following: straight well section, kicking-off section, and stabilizing section. The stabilizing drilling passes through the floor of Coal Seam no.15 for approximately 30 m at an inclination angle of 70° to ensure accurate measurement of the gamma value, gas measurement value, and other characteristic parameters of the target coal seam bottom and floor using a simple gesturing instrument. The pilot hole is sealed by backfilling it with 42.5 grade Portland cement up to the well section with an inclination of about 25°, and the cement slurry has a specific gravity of 1.6–1.7 g/cm3. As the well deviation angle increases, the azimuth angle of directional and composite drilling becomes more stable, particularly when the well deviation angle exceeds 25°, resulting in a smaller azimuth drift^29^. This stability is beneficial for the subsequent inclined side-tracking in the main wellbore's second well section. The pilot hole and main borehole design trajectories are shown in Fig. 6.

Significant data has been obtained through the pilot hole design and the actual drilling of Well-Q. This dataset is pivotal for precise trajectory control in Coalbed Methane (CBM) exploration. The acquisition process relies on several methods, including real-time drilling natural gamma logging for gamma values of marker layers, and downhole gas logging for coal seam gas characteristics. The examination of cuttings recorded in real-time during drilling operations further aids in the identification and differentiation of these marker layers.

**Figure 7:**
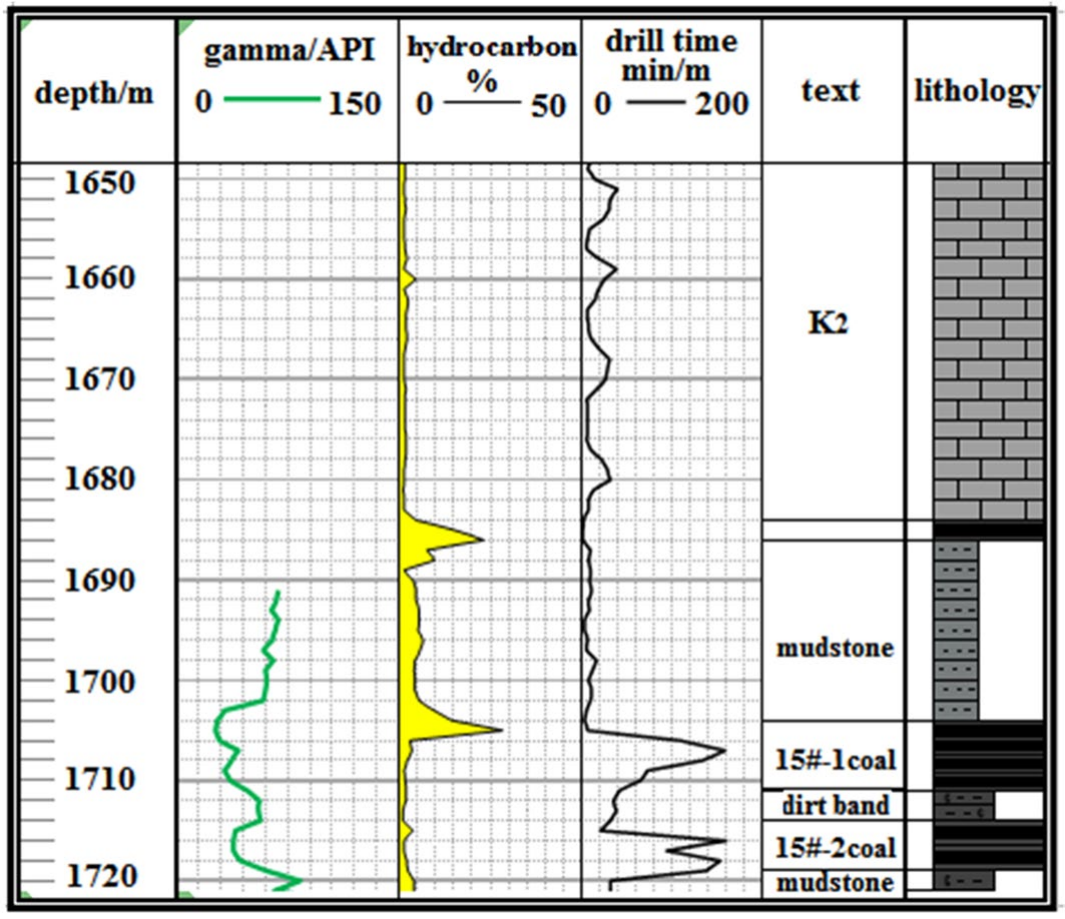
Characteristic parameters and lithology map of the marker layer, target, top, bottom layer.

The critical information gleaned encompasses the identification of the K2 marker bed, the longitudinal stratification of the target no.15 coal seam, as well as the lithological composition, gamma values, and gas-bearing attributes of the upper and lower rock layers. These specific parameters are thoughtfully presented in Fig. 7, establishing a robust foundation for the meticulous control of trajectory and the rational design of the landing point within the target coal seam. This dataset also serves as a valuable point of reference, ensuring the seamless execution of the horizontal drilling phase within the coal seam. Consequently, these findings play a pivotal role in enhancing drilling efficiency, ultimately culminating in the realization of efficient drilling objectives.

### The effect of two-dimensional resonance method

**Figure 8:**
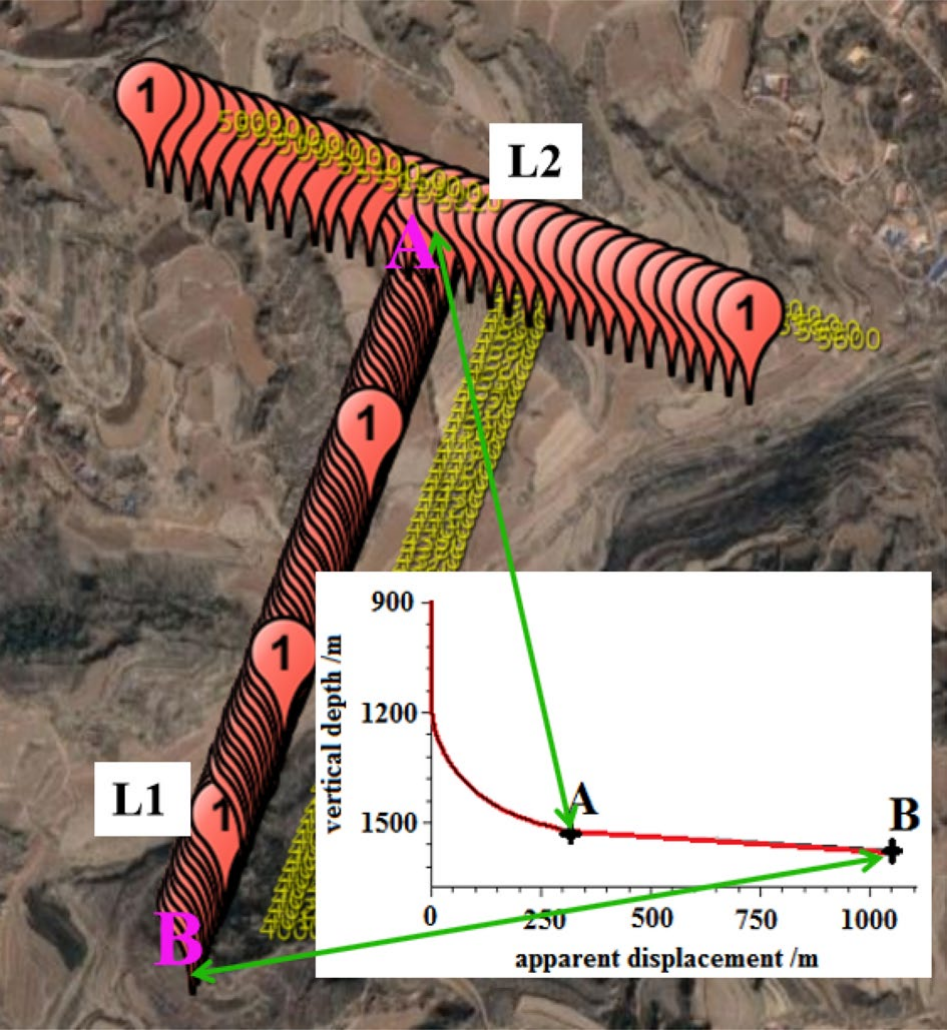
Two-dimensional resonance exploration layout points.

The horizontal section's overall drilling azimuth in the target coal seam is 200°. To identify minor faults in the coal seam azimuth direction, measurement points are arranged every 10 m from the landing point A to the final target point B along the 200° azimuth direction. Additionally, one exploration point is set every 20 m across the azimuth line perpendicular to the landing point A and 200° azimuth direction. Furthermore, exploration points are arranged 300 m along both sides of the landing point. Figure 8 shows the specific layout of the exploration points, where Line (L1) represents the 711 m long horizontal well section of the target coal seam in the 200° azimuth direction. Meanwhile, Line (L2) represents the 600 m long vertical section between the landing point A and L1. The obtained data from these exploration points are crucial in detecting potential faults and ensuring smooth drilling of the horizontal section of the coal seam. ultimately leading to improved drilling ratios and more efficient drilling.

**Figure 9:**
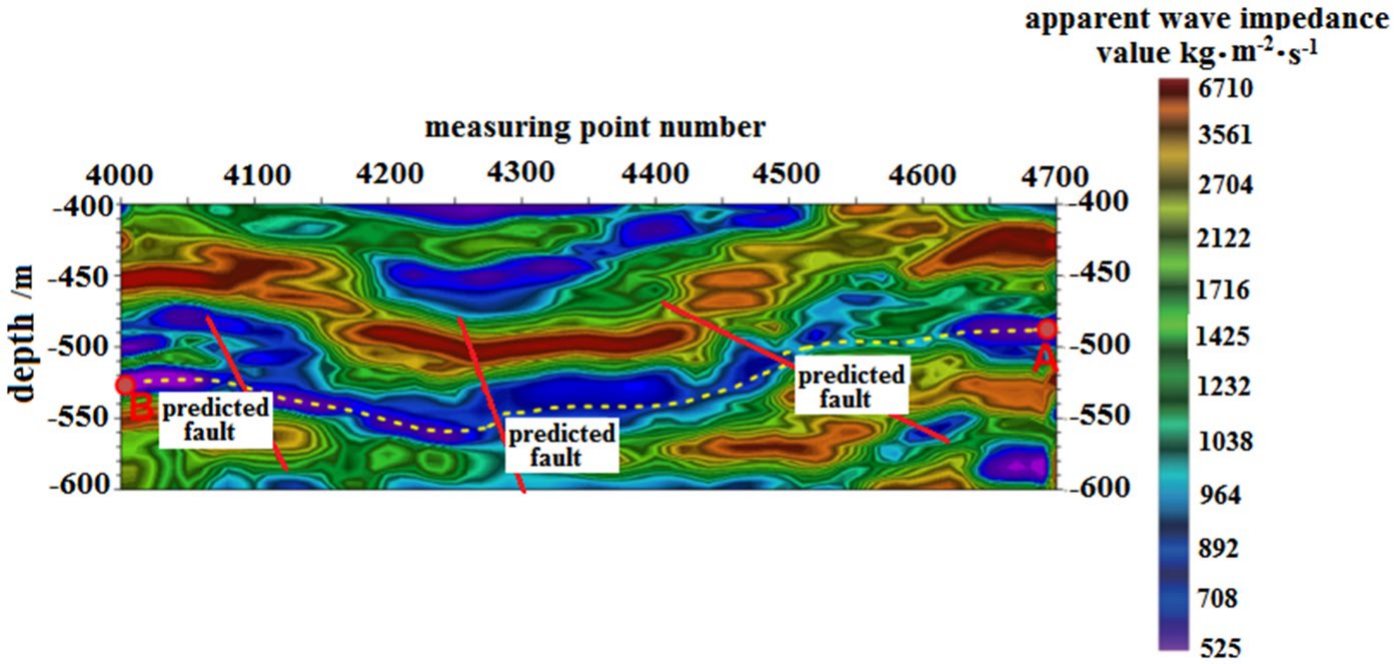
Design of horizontal section trajectory resonance exploration inversion profile.

Figure 9 shows the seismic frequency resonance inversion profile. The trajectory of the designed horizontal section coincides with the ground position of L1, with the no.4700 measuring point located at the ground projection position of the A target point, and the no.4000 measuring point located at the ground projection position of the B target point. Based on the interpretation of seismic frequency resonance line L1 profile, it is observed that the burial depth of the coal seam on the horizontal well section from target A to target B of the no.15 coal seam in the direction of 200° azimuth is shallow in the northeast and deep in the southwest. The overall trend of the burial depth of the coal seam indicates a shallow-to-deep trend. Furthermore, three small faults are expected to be encountered while drilling along this azimuth direction, located at no.4700, no.4280 and no.4096 measuring points, respectively, with a fault distance of approximately 5–10 m.

**Figure 10:**
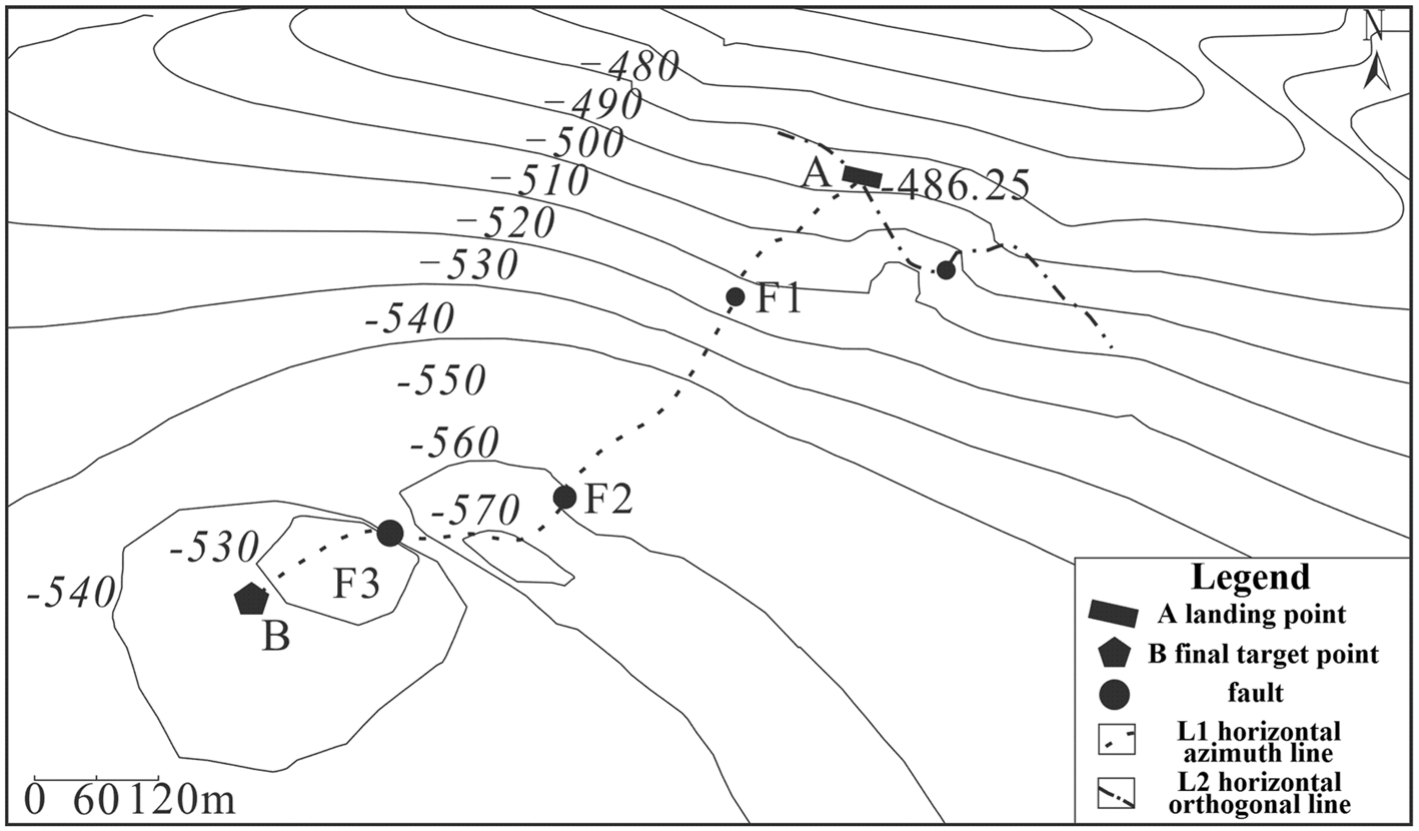
Contour map of fault points in the horizontal section.

The contour map of fault points found in the horizontal section is displayed in Fig. 10. This map serves as a useful tool in guiding the vertical depth control of the horizontal section track.

To ensure that the drilling trajectory is within the target coal seam and to prevent any reduction in drilling ratio caused by the faults, it is necessary to optimize the well trajectory prior to drilling. Each fault point must be considered as a target point and their relative coordinate positions are presented in Table 3.

**Table 3:** Target and fault points spatial location coordinates.

X	Y	Vertical depth H/m	Instruction
4 072 985.89	408 269.51	1 525.92	A target point
4 072 478.03	408 072.02	1 551.47	F1 fault
4 072 273.75	407 994.56	1 589.30	F2 fault
4 072 101.65	407 929.29	1 577.51	F3 fault
4 072 011.00	407 895.00	1 576.53	B target point

**Figure 11:**
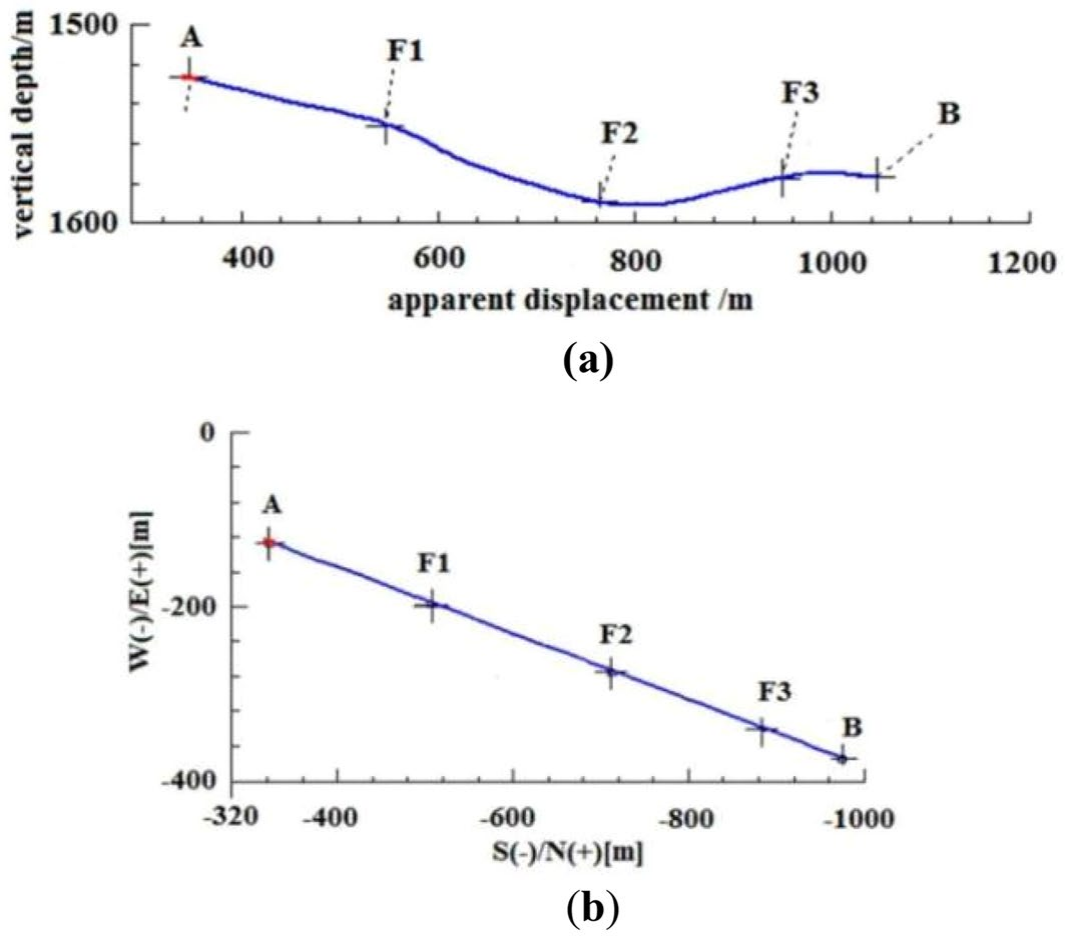
optimized well trajectory for drilling reservoir section. (**a**) vertical section, (**b**) horizontal projection section.

Resonance exploration data is utilized to adjust the trajectory parameters every 10 to 20 m during the actual drilling process. This is before exploring the coal seam behind the fault following reasonable adjustment of the parameters. This method is simple and minimizes the length of the non-coal section during the coal chasing process after drilling through the fault. Based on the coordinate position of each target point, the design of the directional trajectory for the third well section is optimized, as shown in Fig. 11.

The optimized design trajectory should be followed during actual drilling, ensuring that the dogleg degree ≤ 4°/30 m required by the management method for safe operations. Across the fault points F1, F2, and F3, the length of the non-coal section for coal tracking drilling was 56 m, 53 m, and 35 m, respectively. The total non-coal section for actual drilling was approximately 144 m, while achieving a drilling ratio of 80% for the target coal seam with an average thickness of 2.06 m. The entire drilling cycle takes approximately 45 days.

### Azimuth gamma application

**Figure 12:**
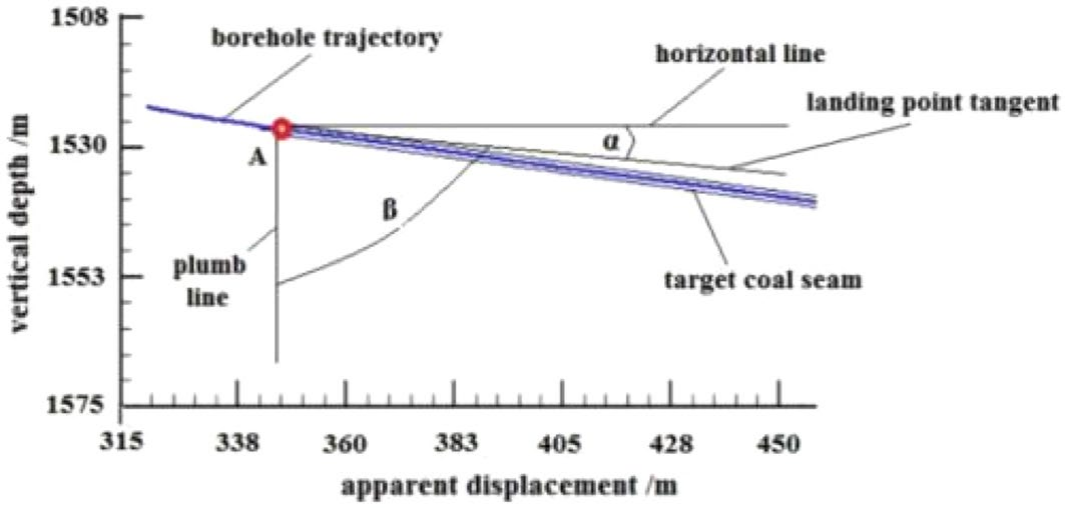
Parameters of the landing site.

By analyzing the azimuth gamma data obtained during the drilling of the pilot hole and using the basic parameters of the pilot hole and formula (1), the apparent dip angle of the stratum near the designed landing point is determined to be α = 6.5°. The parameters of the landing point are shown in Fig. 12, and the deviation angle of the actual main borehole trajectory of the second well section at the landing point β should be controlled at around 83.5° to ensure that the drilling ratio along the coal seam of the third well section is achieved and to reduce the frequency of directional trajectory adjustment.

**Figure 13:**
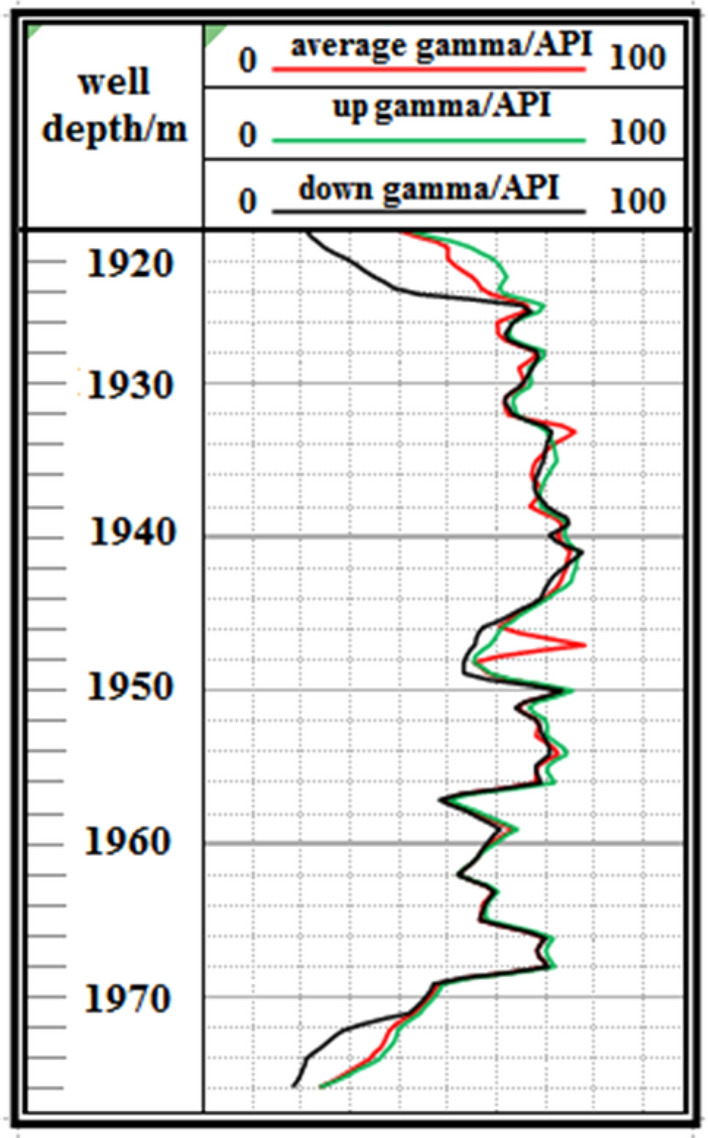
Non-coal seam section azimuth gamma logging curve crossing fault F_1_.

**Figure 14:**
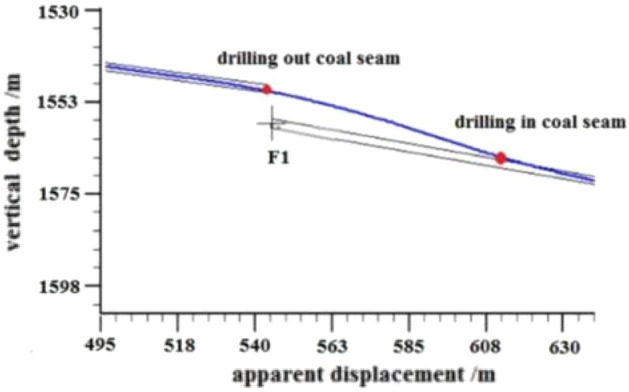
Actual drilling trajectory of fault F_1_ in pursuit coal.

During the drilling of the third horizontal section of Well-Q, a combination of Two-dimensional resonance exploration results and azimuth gamma logging while drilling technology was used to guide rapid coal tracking during the drilling of three faults. The process for each fault was as follows:

F1 Fault: The logging curve in Fig. 13 indicates that the F1 fault caused the drilling track of the 1920–1976 m well section to be drilled out from the coal seam roof. Geological logging revealed that the rock debris returning out of the hole bottom contained a large amount of mudstone. Based on the Two-dimensional resonance exploration inversion (Fig. 9) and fault contour (Fig. 10), the coal seam was traced by drilling with deviation correction through the lowering of well deviation. The actual drilling track during the pursuit of coal process is shown in Fig. 14.

**Figure 15:**
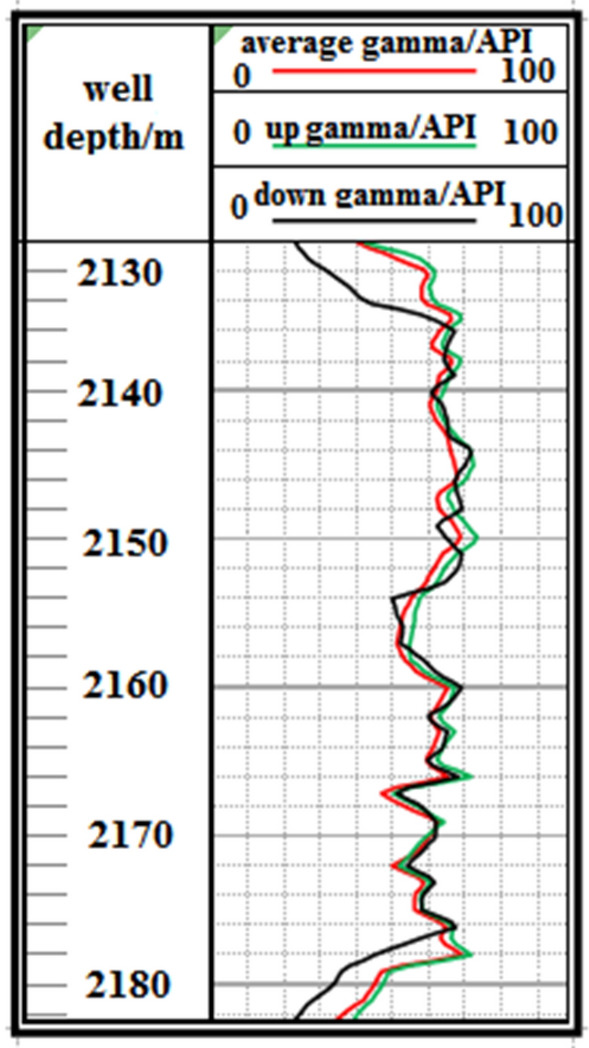
Non-coal seam section azimuth gamma logging curve crossing fault F_2_.

**Figure 16:**
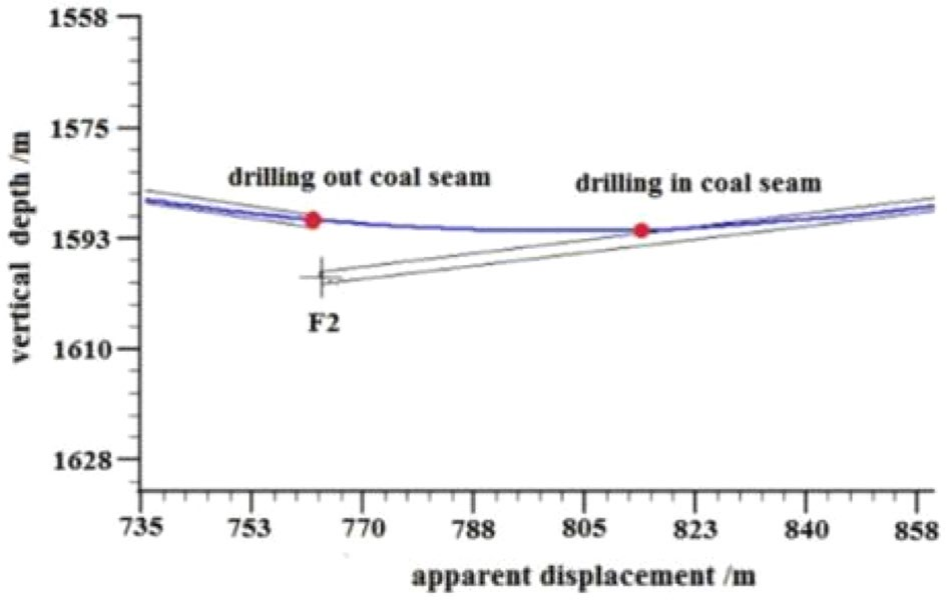
Actual drilling trajectory of fault F_2_ in pursuit coal.

F2 Fault: The logging curve in Fig. 15 shows that the F2 fault caused the drilling trajectory of the 2130–2183 m well section to be drilled out from the coal seam roof. Geological logging revealed that the rock debris returning out of the hole bottom contained a large amount of mudstone. Based on the Two-dimensional resonance exploration inversion (Fig. 9), the back fault block of F2 fault in the direction of drilling trajectory of F2 fault shows a tendency of coal seam incline, so directly using lowering deviation correction drilling to trace the coal seam is not feasible and increases the length of the non-coal seam section. Therefore, the coal seam was pursued by increasing well deviation and rectifying drilling. The actual drilling track during the pursuit of coal process is shown in Fig. 16.

**Figure 17:**
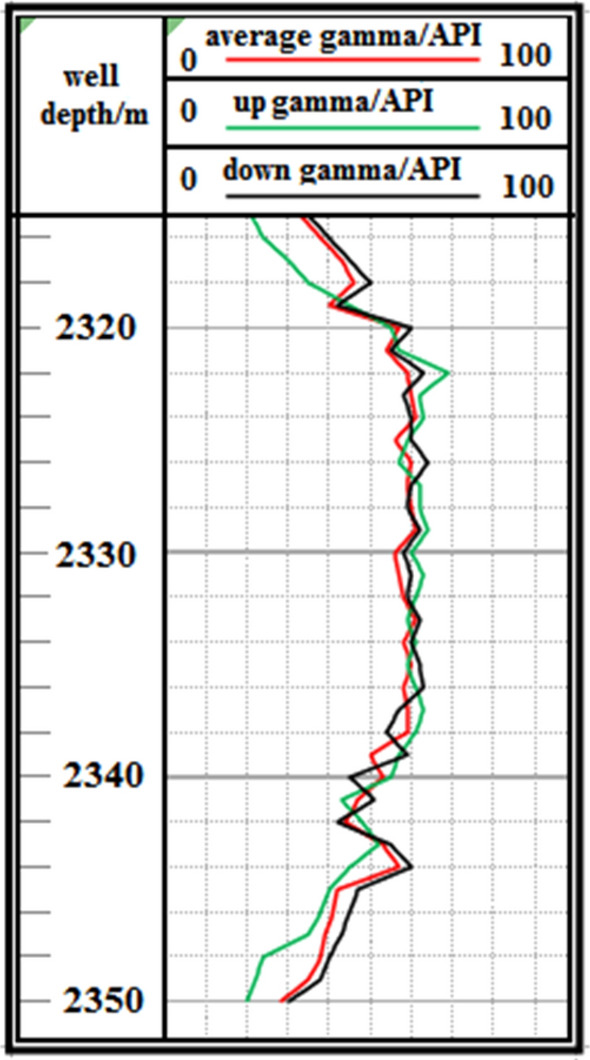
Non-coal seam section azimuth gamma logging curve crossing fault F_3_.

**Figure 18:**
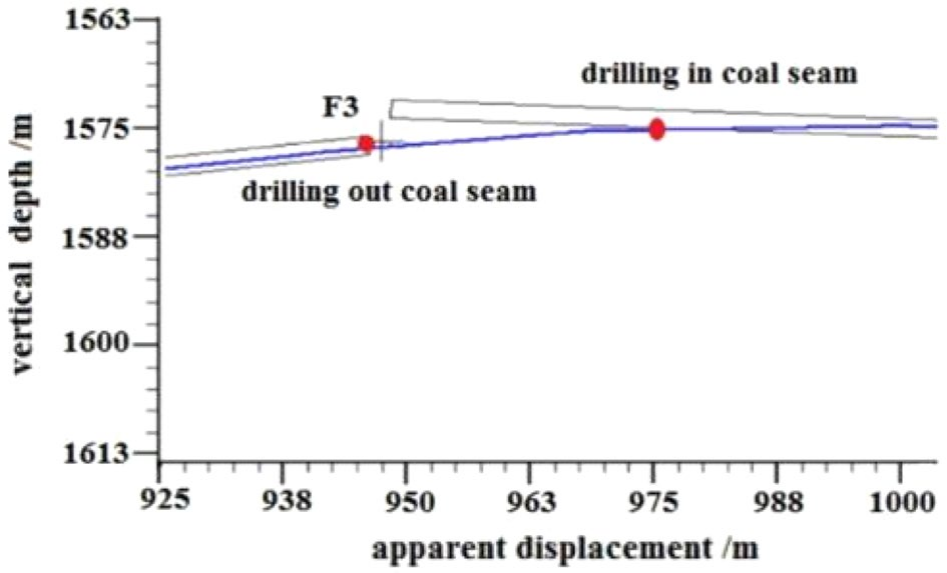
Actual drilling trajectory of fault F_3_ in pursuit coal.

F3 Fault: The logging curve in Fig. 17 shows that the F3 fault caused the drilling trajectory of the 2315–2350 m well section to be drilled out from the coal seam roof floor. Geological logging revealed that the rock debris returning out of the hole bottom contained a large amount of carbonaceous mudstone. Using formula (1), the coal point well inclination angle was calculated as 96°. Based on the Two-dimensional resonance exploration inversion (Fig. 9) and fault contour (Fig. 10), the coal seam was pursued by slowly lowering the well inclination and correcting the deviation. The actual drilling track during the pursuit of coal process is shown in Fig. 18. The well inclination angle was 91° upon returning back to the coal seam, after which drilling along the coal seam was continued normally.

## Conclusion

In conclusion, for the exploration block of CBM, the combined use of pilot hole drilling, two-dimensional resonance exploration technology, and azimuth gamma logging technology has proven effective in controlling the drilling of short-radius horizontal sections along the seam and ensuring the coal seam drilling ratio. Two major points can be drawn from this:

1. The two-dimensional resonance exploration technology detected the development of micro faults in the horizontal section of the drilling, enabling trajectory optimization before drilling. The azimuth gamma logging while drilling technology monitored the current drill bit drilling horizon in real-time, ensuring timely and accurate well trajectory adjustment.
2. The comprehensive use of these technologies has led to a 20% improvement in the coal seam drilling ratio and a 25% reduction in drilling cycle time in tested short-radius wells in the new exploration and development block-W in Qinshui Basin. This provides technical experience for low-cost exploration and development of CBM in new blocks.

## Data Availability

The datasets used and/or analysed during the current study available from the corresponding author on reasonable request.
